# Respectful maternity care experiences of South Asian refugees in the US: a qualitative study

**DOI:** 10.3389/fpubh.2025.1613249

**Published:** 2025-08-01

**Authors:** Denise C. Smith, E. Brie Thumm, Nai Chieh (Geri) Tien, Katherine Kissler

**Affiliations:** ^1^College of Nursing, University of Colorado Anschutz Medical Campus, Aurora, CO, United States; ^2^Asian Pacific Cultural Development and Wellness Center, Aurora Mental Health and Recovery, Aurora, CO, United States

**Keywords:** maternal health, refugee, language interpretation, United States, maternity care, qualitative research

## Abstract

**Introduction:**

Immigrants and refugees giving birth in the United States face challenges in receiving high-quality maternity care. The purpose of this study was to understand the experiences of recent refugees from ethnic communities displaced from southern Asia and resettled in the United States.

**Materials and methods:**

The qualitative study used focus group discussions with three refugee communities who have given birth since resettlement in the United States. Using thematic analysis, we applied the concepts of respectful maternity care to identify themes.

**Results:**

Five themes emerged from the analysis: (1) interpersonal caring, (2) flaws in US maternity care are amplified for refugees, (3) multidimensionality effects knowledge, preferences, and expectations, (4) complexity of the US health system combined with unfamiliarity contributes to lack of confidence, and (5) problems with language interpretation.

**Discussion:**

The identified themes can inform specific, actionable policies and programs that improve care for immigrant and refugee communities including investment in nursing care, implementation of multilingual doula care, improvements in language services, and robust childbirth education.

## 1 Introduction

Maternal health outcomes in the United States are inexcusably poor and characterized by racial, ethnic, and geographic disparities. Maternal mortality for birthing individuals of color is 2–3 times more likely compared to their white counterparts (1). Racially and ethnically minoritized communities also experience disproportionately high rates of adverse maternal health outcomes (2), including higher rates of preterm birth, mental illness, and maternal mortality compared to native born individuals (3). A study of the social determinants of health by Erikson and Carlson found that birthing individuals who immigrated to the United States were 71% more likely to experience maternal morbidity than their predominantly White non-immigrant, economically-secure, highly-educated counterparts (4). Newcomers to the United States, that is recent refugees and immigrants, are a particularly vulnerable population. Newcomers are affected by intersecting social determinants of health, including economic and political disenfranchisement, as well as racial and ethnic minoritization. Research focused on the perinatal care needs of refugees and immigrants in the United States context is therefore an important component of improving maternal health outcomes in the United States.

Refugees and asylum seekers represent a large portion of the perinatal population in the US with unique challenges engaging with maternity care. In 2022, 23% of the 3.5 million births in the US were to foreign-born women, many of whom are immigrants and refugees (5). Immigrants and refugees experience limited access to perinatal care, stemming from structural, organizational, social, personal, and cultural barriers, which contribute to adverse maternal health outcomes (3). Barriers to receipt of quality care include language, discrimination, and lack of cultural competence (6). Evidence indicates that even in circumstances where complications in pregnancy could be effectively managed, women may hesitate to seek care if they have faced disrespectful treatment in the past, and they may also discourage others from seeking care (3, 7).

Individuals from Myanmar (Burma), represent a large and diverse portion of refugees throughout the world and in our geographic region of the United States (Denver, Colorado). Myanmar is a multi-ethnic country of 55 million people rife with internal conflict since achieving independence as a British colony in 1948. Over a half-century of civil wars in Myanmar has resulted in over 3 million internally displaced people and an estimated 1.4 million externally displaced people counted by the United Nations as refugees (8). An estimated 5,000 people from Myanmar, spread across multiple ethnic groups, have been resettled in metropolitan Denver over a 15-year period. Among those resettled in Denver are people representing multiple ethnic groups–including Rohingya, Karen, and Burmese Gurkhas. Over 80% of resettled peoples in Colorado are between the ages of 18–60; many of whom are young adults of childbearing age (9).

Given the specific risks facing refugee populations and the documented barriers to accessing quality care, there is a critical need to develop targeted interventions that promote respectful maternity care for communities like the Rohingya, Karen, and Burmese after resettlement in the US. Adoption of respectful maternity care (RMC) is proposed as a strategy to improve outcomes across populations, particularly for those experiencing health disparities. Respectful maternity care (RMC) is broadly defined as a philosophical approach to care with resultant skills and behaviors by birth practitioners that are person-centered (10). RMC “emphasizes the fundamental rights of women, newborns, and families, and that promotes equitable access to evidence-based care while recognizing the unique needs and preferences of both women and newborns” (11). Studies support positive associations between receipt of respectful care and clinical outcomes (12, 13). Most recently in the US, the Agency for Healthcare Research and Quality (AHRQ) introduced RMC as a key component of patient safety culture and is actively supporting investigation of RMC (14). Researchers have identified 12 domains of RMC, which include providing information and informed consent, maintaining privacy, and competent personnel (see Table 1 for full list of domains) (7, 10, 11, 14). Researchers studying the experiences of RMC of recent arrivals to the US have identified health care providers’ behaviors inconsistent with RMC. Example behaviors include criticizing patients’ choices regarding family structure and procreation (15). However, there is little known literature about how systems factors, such as routinized policies and procedures, contribute to RMC for newcomers.

**Table 1:** Domains of respectful maternity care (7, 10, 14).

Domains of respectful maternity care
Free from harm and mistreatment
Maintaining privacy
Preserving dignity
Prospectively providing information and informed consent
Access to family and community support
Enhancing the physical environment and resources
Providing equitable care
Effective communication
Respecting choices that strengthen a woman’s capabilities to give birth
Competent personnel
Effective and efficient clinical care
Continuity of care

To better understand how system-level factors contribute to RMC for immigrants and refugees, we conducted a qualitative study to (1) explore the birthing experiences of South Asian immigrants and refugees, (2) identify what constitutes RMC for this unique population, and concurrently establish and sustain relationships with communities to inform the entire research process.

## 2 Materials and methods

### 2.1 Methodological orientation and theory

This study is the first portion of a larger project that seeks to identify and prioritize interventions that foster RMC among socially vulnerable communities. Employing thematic analysis qualitative research design, this part of the study utilized focus groups to explore participants’ real-world experiences which will subsequently be used to generate actionable insights grounded in both theory and practice (16, 17). In this study we investigated community perceptions of care received during pregnancy and childbirth among refugee communities after arriving in the US acknowledging the power differential between recent arrivals to the US and the health care system and researchers, the project was developed and implemented using principles of community based participatory research (CBPR) (18). CBPR incorporates an emancipatory lens, and by nature is reflexive and interpretative inquiry (19). We applied the following principles of CBPR: (a) community as unit of identity, (b) build on strengths and resources within the community, (c) facilitate collaborative partnerships and power sharing that attend to social inequalities, (d) integrate and achieve balance between research and action, and (e) emphasize problems of local relevance (19–21). Using CBPR, we aim to lay the foundation for generating emancipatory knowledge that identifies by whom, about who, and for what purpose knowledge is generated. Appropriate ethics approval was obtained (COMIRB #23-1459).

### 2.2 Research team

The team was created as a partnership between researchers and members of a community organization. Team member skills include mental health service delivery, maternity care research, community-based participatory research, and qualitative research methods. The primary researchers identified their positionality; all are practicing clinicians who provide either reproductive or mental health care for refugee populations (three certified nurse-midwives with research degrees and one mental health care provider with a clinical doctorate). All identify as women.

The research team engaged in reflexive examination of their professional and social positions. Professionally, the team brought a perspective on health care delivery as health care providers. Their clinical practice, situated within biomedical institutions yet aligned with principles of holistic care, informed their understanding of care delivery. During the study period, the team provided clinical care to individuals with medical and social risk and acknowledged both the necessity of medical intervention and the complexity of the care environment. They also recognized that overuse of interventions and medical patriarchy largely characterize labor and birth care in the US. The researchers recognized their limited familiarity with the cultural practices and birth-related expectations of South Asian communities. These roles and identities influenced how the team collected and interpreted the data.

Attention was given to relational dynamics between the research team and participant communities during both the interviews and data analysis. Differences in race, ethnicity, education, and social class were recognized as influencing the research relationship. Clinical experiences of the research team suggested that immigrant patients can demonstrate high levels of deference to health care providers, which may also influence interactions within the research setting. The team considered how this dynamic might manifest as compliance or appeasement in research encounters. Hence, the use of a focus group with community based, same-gendered, health navigators-as- language interpreters was selected as a strategy to shift the group power dynamics from answering to the researchers to talking among themselves.

Critical feminist theory served as a theoretical lens informing the study, influencing the researchers’ attention on power, gender, and relational dynamics. At the same time, the researchers acknowledged the value of diverse epistemological perspectives and sources of knowledge beyond this framework. Importantly, data collection occurred during a period of political change (i.e., immigration policy), which was considered relevant to participant narratives. Reflexive engagement continued throughout the analysis and writing process, with attention to shifting assumptions and contextual influences on interpretation.

The community partner organization is a not-for-profit, community-based center that provides mental health services, education, and health care navigation tailored to the needs of Asian refugees and immigrants in an urban community in the Mountain West region of the United States. Two leaders in the center and three community navigators, who are also certified interpreters, participated in the research, including in the design of the research, conduct of focus groups, and member checking of data analysis. The full team met several times prior to conducting the focus groups to determine which language groups and individuals to include in enrollment, how to structure (location, timing, hospitality, etc.) the focus groups, and develop the focus group facilitator guide and key questions, The community navigators were present and engaged with the focus groups and afterward member checked the data analysis. One of the center leaders (G. T.) co-wrote the manuscript.

### 2.3 Participants and setting

The community where the study was conducted is racially, ethnically, and culturally diverse. An estimated 24% of the childbearing population reports a preferred language other than English or Spanish, with over 40 languages represented, including 20 different Asian languages (22). Common Asian languages included Burmese, Nepalese, Rohingya, and Farsi/Dari. The metropolitan area where the study was conducted has been a primary community for refugee resettlement over the last 40 years and is now home to thousands of people forced to flee Myanmar. Often these individuals from Myanmar spent considerable time living in refugee settlements in bordering Thailand before moving to the US.

Our community partner identified three populations based on spoken language that best represented the child-bearing individuals engaging with services at the community organization’s center. Participants were purposively sampled from Karen-, Karenni-, and Nepali-speaking language communities to maximize representation of current refugee populations in this geographic area. Participants were eligible if they had recent experience receiving maternity care in the United States (within the last 3 years), were born outside of the US, came as a refugee, and identified Karen, Karenni, or Nepali as their first language. Participants were approached by community-based patient navigators and invited to attend the group if they met criteria and had interest in participation. It was not required that study participants receive services from the community organization, therefore it was not known if study participants engaged in the services (e.g., mental health services, language classes) provided by our community partner.

### 2.4 Data collection procedure

We conducted three focus groups consisting of 5–6 participants, 2 members of the research team, and a language interpreter. All focus groups were conducted in-person at the center of our community partner in a room typically used for staff meetings and education. Co-located childcare and compensation for time and travel were provided for participants. Each session lasted 3-h and included a shared meal from a local restaurant selected by center leaders. Each focus group discussion was dually-moderated: one researcher served as the primary moderator and focused on keeping the discussion moving openly and freely and the second researcher’s role was to ensure that all topics were addressed. Researchers have experience with qualitative research and employed a researcher-as-instrument technique, acknowledging the experience and lens of the researchers as a tool for data collection and analysis (23). A semi-structured interview guide was developed with the community partner and reviewed by interpreters ahead of data collection. The primary questions were developed from the twelve domains of respectful maternity care and aimed at understanding “What behaviors, policies, or practices have you experienced in maternity care in the United States that made you feel respected or disrespected?” and “What can health care providers (nurses, midwives, physicians) do to provide more respectful care for people in your community during pregnancy?” Additional probes solicited information related to each of the domains of respectful maternity care, participants’ friends and family perceptions about maternity care, and examples of potential changes to practice that relate to each domain. Focus groups were facilitated freely, balancing the questions in the interview guide and the topics of highest importance to the participants. The full interview guide is available in the appendix.

#### 2.4.1 Translation and interpretation

Each focus group was held in the primary language of the respective group (Karen, Karenni, Nepali) and interpreted in real-time, after every few sentences, to English by a certified interpreter of the respective language group. While some participants had English language proficiency, not all members did. In addition, it was the desire of the researchers that participants be able to speak freely and easily about their experiences in the language that was shared among them and most familiar. The research team had no language proficiency in Karen, Karenni, or Nepali. Language interpreters were also members of the community and served as community navigators, who connected members of the community to physical and mental health and social resources. Audio was recorded and the English audio auto-transcribed using Zoom®. Each English transcription was cleaned and reviewed for accuracy with the support of the original interpreter to resolve language questions using recorded audio in the primary, non-English language. Then each transcript and audio recording were reviewed and validated by a second certified interpreter for accuracy of the initial interpretation (24). Lastly, pull out quotes were edited for English language grammar, primarily to aid in readability.

### 2.5 Qualitative analysis

Qualitative data were analyzed thematically. Each transcription was synthesized by the research team in Atlas.ti (web version 25) using an iterative approach (25). Qualitative analysis commenced with the first focus group and was conducted simultaneously with data collection for the subsequent two groups. Initial key themes were incorporated into probes in later focus groups. First, three of the researchers read and immersed themselves in the transcripts and in the ten domains of RMC. Then, two researchers open-coded each transcript and generated initial codes (16). After initial independent coding, the research team refined codes to develop, review, and refine emerging themes. Ultimately, the final themes were defined and named with consideration of the RMC domains. Rigor was maintained by strategies including data immersion, use of multiple reviewers, creating an audit trail, frequent team member meetings for peer debriefing, and ongoing reflexive activities, member checking, and triangulation between three focus groups. The emergence of new themes was monitored across focus groups and found that by the third group, no substantially new themes were identified, suggesting that thematic saturation had been reached.

## 3 Results

Study participants included 15 individuals, ranging in age from 20 to 37 years old, with children ranging in age from 3 months to 14 years old. All participants gave birth in the US recently, a few participants had also previously given birth outside the US, mostly at a refugee relocation site in south Asia.

Five themes emerged from the analysis: (1) *interpersonal caring*, (2) *flaws in US maternity care are amplified for refugees*, (3) *multidimensionality effects knowledge, preferences, and expectations*, (4) *complexity of the US health system combined with unfamiliarity contributes to lack of confidence*, and (5) *problems with language interpretation.*

### 3.1 Interpersonal caring

*Interpersonal caring* reflects the verbal and physical expression of relational and emotional aspects of care between a patient and health care provider that recognizes and responds to their needs, either spoken or unspoken (26). Focus group participants expressed valuing this type of caring and stated that most of the individuals they encountered within the health care system (e.g., nurses, midwives, physicians) demonstrated caring. Participants mentioned that staff smiled at them, were kind to them, and used encouraging language.

I remember when I push[ed] (during second stage labor) with my first daughter, I liked the midwife. I liked her words. She said, “you’re a strong woman you can do this!” I became strong all of a sudden. I pushed two to three times, and my baby came out. I felt very weak when she didn’t encourage me. Right after she started to encourage me, maybe my heart became stronger, I pushed harder. — Karen Participant

The participant’s acknowledgement of the importance of verbal encouragement from providers supported her ability to give birth. Her observation indicates that the actions of providers, in this case, verbal encouragement, are important components of receipt of respectful care.

Many participants reported that gentle touch from providers was appreciated, although a few mentioned that providers were too rough with their infant and with themselves when they were in pain, thus perceived as a lack of caring. Participants stated that providers asked explicitly for permission and consent prior to exams, procedures, or treatments. They generally felt their autonomy was maintained and that their privacy was ensured during care. Participants said that during their hospitalization they valued being cared for in an intimate way through the meeting of physical needs. They specifically mentioned that food and hygiene supplies were provided and that nurses helped them to the bathroom postpartum. Participants expressed a desire to be supported with human presence and not being left alone.

…things I like about having a baby here is that they take good care of you like your mom. Close, like your mom is close to you. — Nepali Participant

The participant described receiving care that felt deeply personal and intimate care that echoed the attentiveness one might associate with a mother. The comparison highlights an appreciation for a style of caregiving that goes beyond routine medical attention but rather emphasizes emotional presence, sensitivity, and the anticipation of needs. Such care is not merely about clinical competence but about relationship, where the caregiver is attuned to the patient’s unspoken needs and responds with warmth, reassurance, and support.

This kind of intimacy in care, where needs are met before being voiced, reflects an appreciation of care rooted in human connection. This implies that maternity patients do not only require physical support, but also emotional connection during a time of physical exposure and psychological vulnerability.

### 3.2 Flaws in US maternity care are amplified for refugees

This theme recognizes the ways in which a flawed maternity care system creates additional challenges for refugee communities. Participants identified several issues with systems of care. These issues, detailed in several sub-themes, are well documented failings with US maternity care across populations but appeared magnified for the refugee community.

#### 3.2.1 Early access to care

For example, participants described significant challenges with being unable to access care in early pregnancy. Many did not learn of their pregnancies until the second trimester, and several had multiple encounters with the health care system in which their pregnancy diagnosis was missed. One patient reported having had surgery and learning soon after that she was pregnant during the surgery.

I think… for me… 14 or 11 weeks is too late. Too late… They say wait until "your… miscarriage period has been passed… There's a high chance of miscarrying before 12 weeks.” If we had been seen from right after 6 or 7 weeks, you have more safety. — Nepali Participant

The participant is expressing the desire for entrance to care in pregnancy is soon after diagnosis of pregnancy rather than late in the first trimester, when the threat of miscarriage is significantly decreased. Like many pregnant patients, they expressed frustration with limited early pregnancy care and desired more face-to-face provider support in early pregnancy, especially when experiencing bleeding or miscarriage symptoms.

Participants similarly reported difficulty knowing when to come to the hospital for labor admission, often feeling uncertain about timing and fearing they might be sent home if they arrived too early. While this uncertainty is common for birthing patients, language barriers, unfamiliarity with the US health care system, limited childbirth education, transportation challenges, and limited social support made it especially challenging for this group.

#### 3.2.2 Communication breakdowns and waiting

Participants expressed dissatisfaction with extended wait times in both outpatient and inpatient settings. In the outpatient setting, they reported delays in getting appointments, long waits in waiting rooms for procedures or labs, and additional time waiting for providers in exam rooms. Inpatient, some felt they were left alone for extended periods without clear communication about their care plan and perceived these delays as reflective of differential treatment based on language, skin color, or refugee status. One participant noted, “[t]hey thought, they can wait, they do not mind waiting” and felt health care staff disregarded participants’ time constraints, such as needing to pick up children or attend other appointments. Poor communication about wait times, often due to language barriers, further exacerbated frustrations. Participants also reported canceled appointments or delayed care when interpreters were unavailable.

They tell you, “Oh, we'll be back in a minute,” and they give you the sign where you can beep if you have some emergency, and you call them sometime you feel thirsty. Sometimes you have to go into the bathroom, and they never come back like somebody will come back in 5, 6 hours like when another shift started, and if you if you call them, they will receive the call, and they say, “Oh, we'll be there shortly.” And then they take half an hour. — Nepali Participant

The participant is describing how consistently poor communication combined with lack of follow through contributes to increasing distrust in the system as a whole. It was not known the circumstances of the providers that led to delays, but it is possible to conceive of precipitating factors leading to delays in care beyond their control and part of the larger systems issues related to inadequate staffing, high volume, or other issues.

#### 3.2.3 Repetition in care

Participants expressed frustration with the repeated questioning during their birth hospitalization, particularly while they were in labor. One participant relayed being asked about pain in labor,

They ask questions like “how are you doing?”… asking me when I have pain. They come in “how are you doing? How’s the pain?” It goes like that. I nod and because then you wait a few hours and then come back. They keep asking that. They [are] not say anything. They just ask, “How you doing? How was the pain?” And it was really painful, how can they help? I hate it when I was in pain, like really pain… Can you lay down? Can you stand up? Can you sit? I was so mad, and they keep me asking. — Karen Participant

The participant reported being asked questions they had already answered prenatally—such as those about food security, home safety, and transportation—and felt disrespected when these were repeated, as if their initial responses had not been heard. They also noted that the Edinburgh Postnatal Depression Scale was administered too frequently and without adequate explanation, leading some to provide inaccurate answers to avoid further questioning. This burden was intensified by the time needed to communicate through an interpreter.

#### 3.2.4 Logistical issues

Lastly, participants discussed practical issues of accessing care. For example, inconvenient or unavailable parking for prenatal visits and labor which is a common challenge in congested medical centers. One participant reported receiving multiple parking tickets, while others reported being late to appointments due to parking difficulties.

[I had a] hard time parking. Yeah. So, like, I mean, I have 2 times ticket. The first one is that when I went in for the delivery, the second was appointments with my-after I have my kid, my baby. So I have like 2 time for ticket for parking. — Karenni Participant

There is a clear mismatch between what people expect from pregnancy care and what is provided. When considering data revealed as part of this theme, it uncovers a current system designed primarily to support a business model, rather than one be responsive to the needs of individuals. Patients desire timely care, ease of access, and effective communication—yet these expectations frequently go unmet. The system appears to be structured to avoid use of time or resources on pregnancies that may result in miscarriage. With the availability of highly sensitive over-the-counter pregnancy tests, many people are aware of their pregnancies as early as 1 week after conception. This gap between early awareness and delayed access to care reveals a systemic failure to align with patients lived experiences and emotional needs during this vulnerable period. These systemic failures contribute to a growing distrust.

### 3.3 Multidimensionality effects knowledge, preferences, and expectations

This theme refers to the interaction of each participants’ unique combination of lived experiences, personality, and culture that informs their specific desires for pregnancy care. Participants expressed a multidimensionality to their identity—a product of a lifetime of experiences which were complex and overlapping — that contributed to their experiences of care. Some contributors to their individual identities included the culture of their family of origin, the local community, their generation (i.e., Gen Z or Millennials), and/or the experience of being a refugee which often included experiences in a refugee camp and with resettlement in the US. While participants generally had similar culture of family of origin, they each applied this to the context of birthing within the US health system in a unique way. There was often an internal tension for participants in how culture and experience affected preferences.

This internal tension manifested in different ways. For example, participants agreed that within their cultures, there are traditional foods served to women postpartum. For some participants, access to traditional foods was very important, but others preferred American foods like pizza or French fries. However, participants consistently requested hot water for drinking and showers be made available postpartum in keeping with cultural traditions.

[With] my first daughter, my mama brought me rice and a soup. It’s not a normal-people soup. It’s for when you give birth and you have to eat soup. I didn’t like it at all. I ordered a hamburger and pizza. I like that food. I don’t know why. — Karen Participant

I told her “I don’t want the cold water,” so I asked her for the cold water to be hot water, she said, "Cold water?” “No, I want hot water.” So she said, “Okay,” and went to get the hot water. And also, the shower put out cold water as well. For us after delivery, we want hot water. So, our parents when we get home they warm the water, so we will shower with hot water, not cold water. — Karenni Participant

In relation to the broader purpose of the study, specifically, how the experience of care can inform system-level changes, the data suggest that providers should avoid making assumptions based on a patient’s cultural background alone. It is insufficient, for instance, to say in a clinical handoff report, “This patient is from Nepal, therefore you should not offer her the hospital menu,” or to automatically omit ice when filling her water jug. The findings indicate that many care decisions were driven by individual preferences rather than cultural consensus or dominant U.S. birthing norms about what patients “should” or “should not” do during labor. For example, while many participants opted for epidural analgesia, particularly during their first labor, others preferred unmedicated births. Similarly, although most participants expressed a strong preference for in-person antepartum and postpartum visits, some valued the flexibility and convenience of telehealth options. These variations emphasize the importance of person-centered care that respects individual preferences rather than relying on generalized cultural assumptions.

### 3.4 Complexity of the US health system combined with unfamiliarity contributes to lack of confidence

This theme reflects the disconnect between traditional health beliefs and the complexity of the biomedically-oriented, medical-technical model of care in the US. The biomedical model relies heavily on the use of technology and standardization of practices that aren’t common across the world. This is particularly evident in a shift away from physiologic labor and increased use of labor induction and increasingly non-existent community based childbirth education programs. Participants recounted stories about their maternity care that reflected a limited information with pregnancy, birth, and newborn care in the US health system that led to communication breakdowns and limited confidence in the care they received.

In addition to a lack of familiarity with the US health system, participants noted health care providers not identifying their lack of familiarity. This resulted in participants not feeling confident to make requests about their care. An example was a participant recounting care after a cesarean birth when a nurse brought her cold water, not hot water as is customary in the participant’s culture, and the patient not initially asking for hot water.

And after she had that surgery, she's so thirsty because her blood came out a lot… and she's asking for water and nurse gave her cold water. She didn't know it herself at that time so, and when the water came she drank it. She’s still thirsty. The nurse went to get the cold water again for her… So, after that she fainted… she slept a couple of days. Then she wake up in 2 days and she noticed that they give her cold water. And then after that she asked for hot water instead. —Karenni participant

The participant is conveying a mental association between consumption of cold water (as opposed to hot water) after birth and suboptimal outcomes (in this case the clinical effects of blood loss after birth). The necessity of only hot water consumption was a commonly held cultural belief in their community. Similarly, the research team understood that the participant’s feeling thirst was a symptom resulting from a postpartum hemorrhage, and it was appropriate to give a patient recovering from a postpartum hemorrhage additional oral fluids. However, the healthcare providers nor the research team (who are also clinicians) made the connection between temperature of the oral fluids and the patient’s desired response to oral fluid administration. The lack of the healthcare team’s understanding of cultural expectations of oral fluid temperate contributed to the participant’s reduced confidence, even if no such association exists. Similarly, several participants across focus groups talked about feeling like the hospital staff had not kept their babies warm enough because they did not use enough blankets. They ascribed adverse sequalae to infants not being covered, as described in the following two quotes:

The midwife delivered the kid, and they did not cover the baby fast. Since they took so long, that’s why three of her kids also have yellow skin as well. They also put the kid under the light. The blue light. So, she felt like they were not helping in the lights (need more light and warmth). So, her kid’s skin is so yellow, so she feels like they need to put more light on it. —Karenni participant

… the way she delivered the baby, the baby was healthy, and everything's good. But in after like 8 hours, my baby's skin became yellow, and she also had a little bit of a runny nose. So, I feel like because of what they did-they took so long to cover her with the blanket. —Karenni participant

These statements revealed the limited exposure of healthcare recipients to the technical environments of healthcare in the US and the healthcare teams limited knowledge of cultural expectations in pregnancy and birth care (e.g., keeping the baby covered with a blanket, even under radiant heat). It would be unrealistic to expect that healthcare consumers would have knowledge or familiarity with highly technical equipment, like radiant heat warmers, or the use of phototherapy to treat hyperbilirubinemia. Yet, more knowledge of these treatments could have increased confidence in the care they were receiving. The stories about blanket use suggested that information about suffocation risks may not have been clearly communicated. Patients also described challenges understanding education provided by the health care team because they could not understand the rationale on infant care. The following quote describes a participant’s experience with lactation teaching:

We go to see our baby doctor, but when I give my daughter the nipple she [doctor] says it’s not right, “when you give your daughter the nipple you have to put the whole thing.” I told my husband “I can’t do that!” Because in our country ladies do not do it like that [lots of laughter]. It’s different. It’s not easy here. You have to hold the baby like that [gestures holding a baby to the side instead of upright]. I don’t know. It’s not right [more laughter]. I’m about to cry. I can’t do it like that. I’m so tired! —Karen

This quote, when considered alongside similar accounts, highlights a persistent disconnect between cultural expectations and the biomedical model of care delivery. If unaddressed, this gap can contribute to poorer health outcomes, increased morbidity, and diminished trust in the health system. While providers may emphasize the urgency of medical decisions, the use of coercive language—such as “if you do not consent, something bad will happen”—is inappropriate and can further erode patient trust. Instead, there is a critical need for tools and competencies that facilitate shared decision-making and bridge cultural and biomedical perspectives.

### 3.5 Problems with language interpretation

This theme highlights the challenges participants faced when accessing accurate and timely interpretation services during their maternity care. Participants in the focus group relied heavily on language interpretation to navigate the health system and communicate with individual members of the health care team. Limitations of interpretation were identified frequently. Participants reported having access mostly to telephone interpretation with occasional in-person interpretation for outpatient visits. A few participants stated that if they had a close family member who spoke English with them, they preferred to have that person interpret for them because they knew that person had their best interest in mind.

Like something where my midwife, or a doctor, says something to me if I do not understand my husband translates for me. Because my husband and me, we are so close. Sometimes the interpreter is confusing. — Karen Participant.

The participant is describing the desire for interpretation from a family member over a service, particularly when they possess some, though limited, English proficiency. And while language interpretation was generally widely available, participants described several instances when an interpreter was not available. One participant said she had a prenatal visit rescheduled after she had already arrived at the clinic because the interpreter was not available. Other participants described not having an interpreter available during their births when they occurred at night.

Sometimes they have a hard time getting an interpreter for her, so they just communicated in sign language. — Karenni Participant.

Participants reported that interpreters often added comments to their interpretation which made them question the accuracy of the interpretation. Participants shared a few stories of egregious behavior by interpreters, particularly when the interpreter was male. Participants reported being told not to ask specific questions of the health care provider, that the provider had already answered the question, or that the health care provider was not interested in the information the participant wanted to share with the health care provider. Participants reported that they shared the names of individual interpreters who repeatedly provided disrespectful service in their community and avoided them when possible. However, as patients, the participants had little control over initiating, terminating, or switching interpreters. They also shared that even when the interpreter was using a common first language, there were dialect shifts among immigrants to the US that limited the interpreter’s ability to fully understand, especially if the interpreter was not located in the US.

When he interpreted in-person, he wouldn’t let me ask questions to the doctor. When I asked or tried to ask questions, he became impatient and mean. — Karen Participant referencing dilemmas with a male interpreter

Pain was so difficult, and all the interpreter was, keep repeating, “What are you saying? What are you saying?” And she was like super mad, and she was saying, “Don't you hear what I'm saying?” I can understand English, although I don't know how to reply back, but when you're with a phone interpreter, they add stuff in the middle-what you are not saying. They just add it, and whatever stuff. — Nepali participant

These quotes illustrate some of the more concerning limitations of interpreter services, particularly when interpreters are based in regions where cultural expectations around gender, autonomy, and self-determination differ significantly from those in the US.

## 4 Discussion

Our study identified both positive and negative experiences among Southern Asian immigrant and refugee individuals who received pregnancy and birth care in the United States. While participants generally felt cared for by health care providers—including nurses, midwives, and physicians—they also reported significant challenges, particularly related to language barriers that adversely impacted their care. These barriers exacerbated systemic issues already present in the broader U. S. maternity care landscape. A lack of familiarity with the US health care system contributed to reduced confidence in both the care team and participants’ own ability to engage in health-related decision-making. The diversity of participant experiences reflected the multidimensional nature of identity, expectations, and needs, revealing system-level gaps in culturally informed communication, trust-building, and explanatory practices. These findings underscore the need for system-level interventions—such as revised policies, protocols, and training programs—that prioritize and preserve respectful, person-centered care.

Among the prior studies conducted on refugee health care, a systematic review by Bradenberger and colleagues describes the “3C model,” which identifies three interrelated, primary challenges for immigrants navigating health systems in high-income countries; confidence, communication, and continuity of care (27). In this study, we found agreement with the body of literature informing the “3C model.” In addition, we propose inclusion of a fourth relevant concept– *caring.* Caring, conceptualized as a relationship between caregiver and patient that is grounded in compassion, dignity, love, attention, and authentic presence, is a central tenet of respectful maternity and nursing care (26). This study identified interpersonal care as the most influential factor shaping patients’ perceptions of respectful treatment during labor and birth. These values are expressed through providers’ actions: addressing physical and emotional needs, sharing information through teaching, and offering a presence that fosters emotional expression (26). Caring manifested in both practical tasks, such as assisting with mobility, and more intangible forms, such as providing calm, supportive companionship. During labor, when individuals may be unable to advocate for themselves, the presence of someone attuned to their needs is especially important. This type of intimate, responsive care is often lacking in the dominant medical-technical model of U. S. maternity care, which prioritizes efficiency and risk reduction over relational aspects of care. As a result, the nurturing, mother-like support described by participants is frequently undervalued or absent, contributing to feelings of neglect or disrespect during a time of heightened vulnerability. For hospitals to embrace caring in this sense, they must prioritize and invest in high-quality nursing care. In the United States, where most births occur in hospitals and there is a persistent shortage or notable absence of midwives and obstetricians, nurses play a vital role. Small actions, such as how a patient is addressed or touched, carry deep meaning in the context of childbirth. Recognizing and supporting these elements is essential to achieving respectful, person-centered maternity care.

Second, specific patterns or preferences for care based on cultural preference across communities were not identified. Rather, the data affirmed the importance of approaches to care that acknowledge the uniqueness of each individual and consider the impact of culture and life experiences on patients’ preferences and expectations. Health care provider training often emphasizes the delivery of “culturally competent” care. While the underlying intent is important, implementation frequently centers on avoiding the imposition of the provider’s own cultural framework, rather than actively engaging with the diverse values and needs of patients. This finding highlights the critical importance of person-centered care—approaches that recognize everyone as a unique person with distinct beliefs, preferences, and expectations for care. Effective perinatal care does not mean offering all patients the same things, but tailoring care to their expressed needs (28). A study on experiences with perinatal care in California presented findings affirming that recent immigrants and women of color are less likely to report receipt of person-centered care (29). Individualized, person-centered care requires health care providers to first *ask*, then *listen*, *understand,* and *attend to* each person’s values and needs, allowing for the adaptation of care to best meet them.

Study participants expressed expectations for care that reflected their evolving identities as individuals living in the US, often referencing rights-based frameworks and asserting, “We live here now,” or “This is what it is like here.” These statements emphasized a shift toward expecting autonomy and respectful treatment consistent with US norms. At the same time, participants highlighted interpreters’ limited capacity to navigate the high-intensity and gendered environment of labor and birth—limitations that were sometimes, though not always, related to the interpreter’s gender. The data suggest that interpreters who are women and who possess familiarity with both US cultural norms and those of the country of origin may be uniquely positioned to bridge both worlds and provide more effective, culturally responsive interpretation during childbirth.

The data supported that language interpretation is multi-dimensional and involves more than exclusively verbal translation. Gaps in interpretation services were particularly evident during labor and birth, which are often hectic environments with shouting, crying, multiple people talking at the same time providing various instructions, and simultaneous verbal and non-verbal communication. Although US government standards for language interpretation emphasize accuracy and reliability (30), our findings revealed shortcomings in interpreters’ awareness of the clinical context (e.g., a birth suite, activity in the room, equipment, people) and in their ability to communicate patient needs in that environment (31–33). Participants dissatisfaction with interpretation services was evident in their expressed desire to have family fill that role, especially when intimacy and proximity were useful for communicating needs, particularly nonverbal (e.g., facial expressions or gestures). Gender differences also appeared to contribute to interpretation difficulties, such as when a male translator was communicating about vaginal and cervical exams. Illustrations from the data included translator lack of familiarity with female-specific care (e.g., “he does not say the right thing”) and gender-related cultural implications (e.g., being told by a male translator in their home country, where women are less empowered, that they were asking too many questions).

Participants described how family members, despite limited English proficiency or lack of medical vocabulary, were able to meet interpretation needs in ways professional services often did not. Participants acknowledge that family members may not be equipped to interpret important clinical content, like informed consent, yet they valued their presence for conveying emotional needs and avoiding the burden of verbalizing every request through an intermediary. This preference, while at odds with prevailing best practices, evidence, and polices for interpretation, was strongly expressed (34, 35). In obstetrics, literature focuses on interpretation of information communicated by the provider to the patient (36, 37). However, there is minimal published data available that conveyed similar information to what was learned in this study-the voice of those giving birth and their explanations for preference of family members to interpret during labor and birth. The results of our study offer a perspective on use of family members for interpretation that requires further examination.

Lastly, our findings are consistent with previous studies based on the experiences of women of color in pregnancy and birth, citing lack of information or incomplete information leading to feeling less cared for or respected during the perinatal period (38). Limited or incomplete information about pregnancy and childbirth and care delivered by the health system (particularly related to technology and equipment) is a factor that limits decision making and reduces confidence in navigating health care. Education may also help bridge the gap between cultural traditions and the biomedical model, or at a minimum, equip individuals with tools for effective self-advocacy in meeting cultural expectations from care providers. This is particularly salient when cultural expectations bring added benefit, such as increasing trust and confidence. Many of the barriers identified—such as limited health system literacy and delayed access to prenatal care—are not unique to immigrant populations but are intensified by language barriers and unfamiliarity with the U. S. health system. These structural limitations inhibit self-advocacy and autonomy in health care decision-making (39, 40).

### 4.1 Recommendations

Based on the results of this study, the next step will be to identify system-level interventions that improve birth experiences for refugee communities. We identified preliminary recommendations based on these findings. First, person-centered maternity care system requires implementing evidence-based practices, discontinuing ineffective ones, and incorporating culturally meaningful care. Doula care has emerged as a promising intervention, yet participants reported no prior engagement and expressed uncertainty about involving non-family community members. Doulas can serve dual roles, offering labor-specific language interpretation and emotional support, with evidence showing improved outcomes and communication across clinical, environmental, and interpersonal domains (41–43). Integration of a dual interpreter and support model has shown early success (44–46), barriers remain regarding reimbursement and the clinical role of non-licensed doulas, though recent Medicaid expansions may offer solutions (47). Our data also indicate that doula care may not require language and cultural concordance to be effective as the doula may explain the health care environment and practices to be more understandable to the family even through use of a third party interpreter. Additional research is needed to determine the features most associated with improved outcomes and satisfaction for the newcomer population.

Secondly, broader system reforms are also necessary, including eliminating wait times and fragmented services, improving language access with gender-aligned, in-person interpreters familiar with childbirth (48), and investing in high-quality nursing care. To implement these recommendations requires systems and processes of care that promote quality patient-provider interactions. Staffing models that enable continuous bedside presence, such as 1:1 nurse-to-patient ratios during active labor can enhance relational care and reduce nurse burnout (49). Building relationships between patients and providers during prenatal care can be accomplished more effectively through implementing continuity of care models (50), thus decreasing the need for patients repeatedly communicate their values and preferences. Finally, community-based childbirth education and group prenatal care can bridge cultural and biomedical knowledge, strengthen preparedness and support, and serve as critical entry points for immigrant and refugee families (51).

### 4.2 Strengths and limitations

As contextually situated qualitative researchers, we found a tension between participant’s belief systems, cultural traditions, health education, and the biomedical model that we were not always able to differentiate. For example, a few participants mentioned that providers left their babies naked and uncovered for a prolonged period which they attributed to later development of hyperbilirubinemia. Maintaining infant core temperature is medically important, however this was likely achieved in these scenarios with a radiant warmer and low temperature is not associated with development of hyperbilirubinemia. Participants did not possess information about radiant warmers and maintenance of body temperature, and therefore it was unclear to us whether use of blankets for an infant is related to a belief system, a cultural practice, or knowledge about physiology and information about use of medical technology to support infant temperature, thus a reflection of disconnects between traditional health beliefs and biomedical models of care.

A major contextual limitation of this study is sampling from a single service area. Based on the communities where data collection took place, participants are likely reporting their experiences with care in just 2 clinical sites. Providers who deliver care in these sites are familiar with providing care to these communities, as it has been a major site for refugee resettlement for the last 15 years. Therefore, the care experiences of these communities in a service area with a significantly greater experience in delivering refugee care may differ greatly than the experiences of communities in the US without refugee-dense populations. Second, interpretation between languages is a limitation that may have limited the researchers complete understanding of data and participant meaning. Further, the sample size was relatively small from just three language groups. While thematic saturation was met in this sample, other communities and individuals may have different experiences not captured in the data. Lastly, the community is served largely by midwifery-led models of care, with the largest concentration of midwifery care in the state (40% and higher midwife-attended birth). Because midwifery care is not accessible in large parts of the country, the study may refer to experiences with providers that are less common.

The study benefitted from a robust amount of data from three different immigrant communities. Additionally, participants were able to share their experiences in their preferred language, and interpreters were experienced with health care and cultural translation between the community and the researchers. Further, this study was strengthened by a community based participatory research approach with active engagement of the community in developing the research questions and methods. The use of the RMC framework to guide the interviews and analysis supported methodological rigor, contextualizing the researcher in a larger discussion about maternity care globally. Further, it supported deeper interpretation of the data by connecting the individual experiences of participants to broader patterns.

## 5 Conclusion

This study explored the pregnancy and birth care experiences of Southern Asian immigrant and refugee communities in the U. S., revealing both strengths and critical system-level shortcomings. Participants generally felt cared for by providers, yet faced substantial language barriers, limited familiarity with the U. S. health system, and challenges with communication that undermined confidence and self-advocacy. These findings reinforce and expand upon the “3C” model—confidence, communication, and continuity—by proposing a fourth concept: caring. Respectful care was most often conveyed through interpersonal actions, underscoring the need for health systems to invest in high-quality, relationship-centered nursing care. Key recommendations include improving interpretation services to be more context-and person-aware, integrating culturally and linguistically concordant doulas, enhancing access and system navigation, promoting continuity of care, and reinvesting in community-based childbirth education. Collectively, these interventions support a more person-centered approach to perinatal care that recognizes individual preferences, cultural contexts, and the value of human presence during birth.

There is a strong need, and desire among many, to improve the quality of maternity care delivered in the United States. Using the domains of RMC, we were able to identify themes that can inform actionable recommendations to improve care. Immigrants and refugees to the US are an increasingly larger community who engage now and will continue to be participants in the maternity care system. This study offers insights into their experiences that can be transformed into meaningful interventions for care providers.

## Data Availability

Raw, deidenitified data supporting the conclusions of this article will be made available by the authors, without undue reservation.
