# Supplementary material for: Respectful maternity care experiences of South Asian refugees in the US: a qualitative study
Source: Front Public Health. 2025 Aug 1;13:1613249. doi: 10.3389/fpubh.2025.1613249 (PMC12355223; doi:10.3389/fpubh.2025.1613249)
Supplement: Supplementary file 1 [file Table_1.docx]

**Supplementary Table. Themes, subthemes, and exemplary quotes**

| **Theme: Interpersonal Caring** | |
| --- | --- |
| *Subthemes* | *Exemplary quotes* |
| Most individuals in the health care system provided personal caring | I wanted to be discharged but they wouldn’t let me because the wound down there was swollen so bad. My nurse was very kind. She took care of my very well. I don’t know why but it may be that I gave birth so easily that they rushed with stitches. I’m not sure, but I don’t want to put blame on anyone.] They try their best, but sometimes they make mistake. I don't want to complain about that. [switches to Karen, Lah Lah translation: I just wanted to share my experience. That’s all.] So no complain. They are nice to me. -Karen Participant |
| Gentle touch from providers was appreciated | I remember when I push[ed] (during second stage labor) with my first daughter, I liked the midwife. I liked her words. She said, “you’re a strong woman you can do this!” I became strong all of a sudden. I pushed two to three times, and my baby came out. I felt very weak when she didn’t encourage me. Right after she started to encourage me, maybe my heart became stronger, I pushed harder. -Karen Participant |
|  | Smile. Hold you. Touch you. Very nice. They show you the smile on their faces. They are very gentle when caring for you and hold you and touch you gently. They are very polite and gentle when they speak to you. -Karen Participant |
|  | They were very gentle when they touched and held my baby. They were not aggressive towards my baby. They had a good heart. -Karen Participant |
| Providers express kindness | Yeah, and she was happy. The midwife- they are very nice. -Karen Participant |
|  | Even if the nurses and doctors had different shifts, they took care of me the same. They were all nice. Doesn’t matter. -Karen Participant |
|  | As for me, when I gave birth to my first daughter, and I see the nurse, they are very nice, for everyone is very nice to me. That's why yeah, I don't want to complain you like to do something like a mistake. I don't want to complain to them because they are nice. Sometime people make mistake. -Karen Participant |
| Providers asked for permission and consent; autonomy and privacy was maintained | They ask your permission too. “Can I touch, can I do like this?” Yeah, they ask for permission. Yeah, they are so nice. -Karen Participant |
|  | I was in so much pain that I didn’t care about privacy. -Karen Participant |
|  | Yes, they did really respect. Yeah, I think everyone is very respectful to us. -Karenni Participant |
| Intimate care through meeting physical needs specifically food and hygiene | ...things I like about having a baby here is that they take good care of you like your mom. Close, like your mom is close to you. - Nepali Participant |
|  | They make you feel better. If you are in pain but you’re a doctor, a nurse, a midwife she ask you something like if they smile, you feel like your pain is better. -Karen Participant |
| Desire for human presence and did not want to be left alone | I was alone in my room… I was very scared and had to call my mom. -Karen Participant |
| **Amplification of the flaws of the U.S. maternity care system** | |
| Parking issues | [I had a] hard time parking. Yeah. So, like, I mean, I have 2 times ticket. The first one is that when I went in for the delivery, the second was appointments with my- after I have my kid, my baby. So I have like 2 time for ticket for parking. -Karenni Participant |
|  | Almost 45 minutes looking for a spot in the parking lot! -Karen Participant |
| Delay in pregnancy diagnosis and challenges accessing early pregnancy and miscarriage care | I think...for me...14 or 11 weeks is too late. Too late...they say wait until "your...miscarriage period has been passed...There's a high chance of miscarrying before 12 weeks,” If we have been seen from right after 6 or 7 weeks, you have more safety. -Nepali Participant |
|  | So, when she got sick, she just thought that she had Covid-19. So, she went to see a doctor at the [clinic]. So, they did a Covid-19 test on her, so she had the sickness of Covid-19, so they sent her back home, but she is still feeling so sick. So, she went to [a different clinic]. So, they did a test on her, a pregnancy test. So, they found that she was pregnant. -Karenni Participant |
|  | I have a little bit different experience with my first daughter. So when I was pregnant like a month, I had a surgery and I had an emergency I had to remove my appendix. So this time and before the surgery, the doctor tested me to see if I was pregnant but the doctor said I’m not pregnant when I had surgery at this time. So after the surgery, I'm missing my period a month and then almost a month and a half, so I go back to- told my husband “Maybe, maybe I'm pregnant.” But my husband said “no, because when, after surgery, you and me were not sleeping together.” So we are not together. So, okay, I'll go back to [Mango House] and the doctor test me again. And then they say, “No, I'm not pregnant.” So okay. So how I missed my period, and I'm not pregnant? So almost like a- almost a 4 month again. And so I go back to the Mango House again and the doctor said, “Oh, you pregnant! Since almost 5 months.” So that’s how. -Nepali Participant |
| Difficulty knowing when to present to the hospital for labor care exacerbated for refugees | So the husband’s not at home, call the ambulance, not long, probably just 5 minutes, I cannot wait. Okay, mom, okay. And then the ambulance come and that took her at hospital. So they have so many questions. “Why don't you come to the hospital?” “You want to give birth in here like that?” -Karen Participant |
|  | There was liquid coming out of me at night. It wasn’t my water. There was blood in it. I went to the hospital at 9:00am and when it was 10:00am they gave me an injection to induce my baby. Starting at 3:00pm, I experienced unbearable pain all night. I was already in pain, and they kept asking me if I wanted epidural. I wanted to give natural birth without epidural. They kept asking me and I was already in so much pain, so I got very irritated. I almost cursed at them like “do you want to die?” -Karen Participant |
|  | “because maybe it’s like your first daughter, you know it take a 10 hour and 11hour. So no, it's not like that. So this is like a hour, 2 hour. Must husband get home and we go to hospital. -Karen Participant |
| Dissatisfaction with extended wait times perceived as differential treatment based on language, skin color, or refugee status | So, she feels like because of the language barriers that’s why they treated her that way. “They don’t know anything.” So, they just like, “Oh, it's like, we don't know anything, so they don’t mind waiting longer,” or whatever they think it was. So that’s what she felt like she experienced. -Karenni Participant |
|  | So, she feels like because of the language barriers that’s why they treated her that way. “They don’t know anything.” So, they just, “Oh, it's like, we don't know anything, so they don’t mind waiting longer,” or whatever they think it was. So that’s what she felt like she experienced. -Karenni Participant |
|  | Okay. So, if they say five minutes, five minutes, it’s no problem, but 30 minutes late, we also have another kid to take care of, pick up kids from school, or we have to do stuff at home as well. So yeah, they take so long to come in and check them. -Karenni Participant |
|  | They tell you, “Oh, we'll be back in a minute,” and they give you the sign where you can beep if you have some emergency and you call them sometime you feel thirsty. Sometimes you have to go into the bathroom, and they never come back like somebody will come back in 5, 6 hours like when another shift started, and if you if you call them, they will receive the call, and they say, “Oh, we'll be there shortly.” And then they take half an hour. -Nepali Participant |
|  | Waiting too long. We have to wait too long. We understand they are busy, so sometimes that’s okay. So sometimes it’s too long- for waiting. As for me, even with the short multiple visits, I understand that they [doctors] are busy with their work, so I had to wait longer. -Karen Participant |
|  | When I was pregnant with the second child, I had to consume a liquid before check-up. So the doctor or midwife told me I had to wait for how many minutes? 60 minutes? I don’t remember. But when the time was up nobody showed up. My husband went up and asked the front desk who was going to ask the nurse and they said they forgot. -Karen Participant |
|  | So for the emergency, I had to wait too long. So I came in at 5pm and the doctor came at 11pm. -Karen Participant |
|  | They don't really say, sorry of the like waiting long hours. I also like have experienced that I waited for one hour of waiting, and she didn’t even say sorry. -Karenni Participant |
| Frustration with repeated questions perceived as poor communication and poor listening and intensified by use of interpreter | They ask questions like “how are you doing?”... asking me when I have pain. They come in “how are you doing? How’s the pain?” It goes like that. I nod and because then you wait a few hours and then come back. They keep asking that. They [are] not say anything. They just ask, “How you doing? How was the pain?” And it was really painful, how can they help? I hate it when I was in pain, like really pain... Can you lay down? Can you stand up? Can you sit? I was so mad, and they keep me asking. -Karen Participant |
|  | They didn’t appear happy. They asked me so many questions that I didn’t know how to answer. -Karen Participant |
| **Multidimensional effects knowledge, preferences, and expectations** | |
| Participants identities were shaped by: culture, being a refugee, being in the United States, generation, etc. | [With] my first daughter, my mama brings me rice and a soup. It’s not a normal people soup. It’s for when you give birth and you have to eat soup. I didn’t like it at all. I ordered hamburger and pizza. I like that food. I don’t know why. -Karen Participant |
|  | After I gave birth, I was so hungry, and my mama brought me sou. Stir-fry vegetables. A little bit, not too much. The soup makes your breast milk. -Karen Participant |
| Culture and personal experience met with the complexity of the health care system | I told her “I don’t want the cold water,” so I asked her for the cold water to be hot water, she said, "Cold water?” “No, I want hot water.” So she said, “Okay,” and went to get the hot water. And also, when I share those shower things, the shower did not- also put out like cold water as well. For us after delivery, we want hot water. So, our parents like when we get home they warm the water, so we will shower with hot water, not cold water. -Karenni Participant |
| Knowledge/experience of birth was limited | I was telling them I have no idea about like I'm having a baby, because I have no idea about like what contractions feel like. If there's like getting a pain like getting a period pain. No idea. -Nepali Participant |
|  | …she was telling us she was not well educated on her side. Some people get educated, but some not. On her side, she was not well educated and prepared. She had the C-section. She had to do it. -Nepali Participant |
|  | because I had the worst experience with information they're not providing for my first boy. When I came to hospital, the checkup, everything- I was preplanned, but I have no idea about contraction or whatever. -Nepali Participant |
|  | when you go to a checkup, they don't explain you about like spotting when you get it. They always say like, “Oh, it just depends on your body. You have like some different experience. Other person have different. You can see there this and that, whatever stop.” But we don't have like good knowledge of like, between what months to what months you have like spotting? It's okay and those kind of things. -Nepali Participant |
| Decision making was individual | She wants to give birth natural, so the doctor comes, the midwife, “Do you want to get the epidural?” I say “No no not yet.” But they tried to- they kept asking me again and again…-Karen Participant |
|  | For my first baby, if I knew that that epidural was that helpful, and relaxing, I should’ve [gotten it] in the beginning without having any pain not wait until like last minute. So that's why, for second baby I put the epidural on. I wanted to sleep.-Nepali Participant |
| **Complexity of the US health system combined with unfamiliarity contributes to lack of confidence** | |
| Participants had limited information (about childbirth) | [W]e have difficulty identifying whether it’s contractions or something going on, … I don't have any idea what contractions are... I was working all day long and I was just thinking, like, maybe “It's just like babies trying to move around. Or maybe she's rotating her head around.” I have no idea so I just stay home, and when I see like discharge, and like more frequent contractions. And then I went to hospital around like 10pm, in the evening. And this is like- my baby was almost out because I was 6 and half open. And I had no clue about what contractions are and what it is they mean. They say it, but they don't have an appropriate way of like which time period we have to worry about the contraction. —Karenni participant |
|  | They told her that 4cm means she must wait a little bit longer before giving birth. She didn’t know how many hours she had to wait, but she knew that it’d take longer. -Karen Participant |
| Participants had differing knowledge and/or beliefs from medical community standard knowledge (biomedical model) | You know with my daughter my water broke but I didn’t feel any pain. At 12:00 AM I experienced a sudden abdominal pain that I couldn’t bear, so I had to get epidural. If I pushed at that time, I’d probably have given birth instantly, but I didn’t push at all. After getting the epidural, I had to wait all night to give birth. -Karen Participant |
|  | She miscarried and when she went to see doctor, they see her like every week, and they also draw her blood a lot. And it's like every time she sees them they took her blood out. So when- after they took out the blood when she get back home she so like she feel like she don't have any energy. So she just lay down. -Karenni Participant |
|  | She feel like it's too early to breastfeed the baby that caused her the pain- the nipple pain. -Karenni Participant |
|  | You can listen to them all the time, like after you have a kid, they give you like cold water. Us- We can’t drink cold water. If you drink cold water... [audio cuts out, can’t hear the end of this sentence] It’s really cold water. So, those kinds of stuff is not like you get to see anything. What is safe. -Karenni Participant |
| Differing understanding hindered or delayed shared decision making | And after she had that surgery, she's so thirsty because her blood came out a lot, and she's so thirsty, and she's asking for water, and nurse gave her cold water. She didn't know it herself at that time so, and when the water came she drank it. She’s still thirsty. The nurse went to get the cold water again for her... So, after that she fainted...she slept a couple of days. She don't know it herself, so she's fainted, so yeah. Then she wake up in 2 days. And she noticed that they give her cold water, so yeah. And then after that she asked for hot water instead. —Karenni participant |
|  | So she said the midwife told her to push when she's not ready. So, as you know, the woman who delivered kids, we know our body, so yeah, the midwife told her to push but she like- she don't feel like pushing yet, but they keep told "Push,” so like- and she just did whatever they told her to do so like the more she’s pushing- she don't want to push- but the more she’s pushing, her kid’s head goes up, which caused her to have a C-section. -Karenni Participant |
|  | [My daughter’s] arms were stuck in there, so she couldn’t come out. So, the doctor pushed on me, and I don’t know what that’s supposed to do. But I feel like they need to ask me first. Yeah, but they didn’t ask me, so they just did whatever they did. And after, like she came out, and then after she was cleaning me or the baby, and she was like, “Yeah, I did that to you because your baby was stuck.” So, I feel like she should’ve asked me first before she did that. She should’ve asked me first before she did. -Karenni Participant |
|  | She was saying like they don’t explain whatever it is like- we'll get that like postpartum depression [screening]. …they ask the questions. Just the general questions we have to ask “You feel about like you ever have feelings of like you might kill your baby? Do you get irritated with your babies?” or whatever, and she was shocked because she doesn't have any feelings, and she questioned that back to the doctor "like you have those kind of feelings? Killing your baby? Like you ever feel like you have to kill your baby, or you get angry with your baby?" Like they cry sometimes, and you don't have anybody to look at, or they those kind of feelings pop up. But you never as a mom, you never have those kind of feelings. But she- those- and, they told her like, “It's just the policies we have to ask everyone.” But maybe they don't have a good explanation of what people might get these things, you randomly asking the question. -Nepali Participant |
| Lack of familiarity with technololgy-heavy U.S. health care system led participants to perceive substandard care. | The midwife delivered the kid, and they did not cover the baby fast. Since they took so long, that’s why three of her kids also have yellow skin as well. They also put the kid under the light. The blue light. So, she felt like they were not helping in the lights (need more light and warmth). So, her kid’s skin is so yellow, so she feels like they need to put more light on it. —Karenni participant |
|  | … the way she delivered the baby, the baby was healthy, and everything's good. But in after like 8 hours, my baby's skin became yellow, and she also had a little bit of a runny nose. So, I feel like because of what they did- they took so long to cover her with the blanket. —Karenni participant |
|  | She has low breast milk. And then she asked for formula at the hospital, and then they did not give it to her anyway. And the baby was crying a lot. -Karenni Participant |
| Provider teaching was perceived as insufficient due to lack of familiarity with the health care system | We go to see our baby doctor, but the lady when I give my daughter the nipple she [doctor] says it’s not right. When you give your daughter the nipple you have to put the whole thing. I told my husband “I can’t do that!” Because in our country ladies do not do it like that [lots of laughter]. It’s different. It’s not easy here. You have to hold the baby like that [gestures holding a baby to the side instead of upright]. I don’t know. It’s not right [more laughter]. I’m about to cry. I can’t do it like that. I’m so tired! —Karen Participant |
|  | No one explained anything. I heard that when giving birth, the child is the hardest. I heard this from my parents, not from the doctors or nurses. -Karen Participant |
|  | The first baby is hard. Okay, it's not easy. But the second baby is easy, but from the nurse, from the doctor they never, ever tell you. -Karen Participant |
| **Problems with Translation and Interpretation** | |
| Some participants preferred a family member to interpret | Like something where my midwife, or a doctor, says something to me if I don’t understand my husband translates for me. Because my husband and me, we are so close. Sometimes the interpreter is confusing. -Karen Participant |
|  | So like me, I don't need an interpreter because my husband he does the English very well, so when I need something, and he translates it for me. But you know I can, you know I can speak a little bit, but it's not much, but like something where my midwife, or a doctor, says something to me if I don’t understand my husband translates for me. -Karen Participant |
| Variation interpreter to interpreter including dialect shifts complicating understanding and variation in knowledge of medical terms | Many interpreters, so many interpreters on the phone, and they don’t understand all of the medical terms. So, if you say something like that, constipation, probably they don't understand. They don’t understand that we say, like, the constipation, they are going to say- then they say something [else] -Karen Participant |
| Unavailability of interpreters led to cancelations or poor communication over night | Sometimes they have a hard time getting an interpreter for her, so they just communicated in sign language. -Karenni Participant |
|  | Sometimes they are not able to get a hold of the interpreter and have to wait for a long time and if there is no interpreter, they did not have a discussion and just sent her back home, and they rescheduled her appointment. -Karenni Participant |
| Editorialized interpreting | Pain was so difficult, and all the interpreter was, keep repeating, “What are you saying? What are you saying?” And she was like super mad, and she was saying, “Don't you hear what I'm saying?” I can understand English, although I don't know how to reply back, but when you're with a phone interpreter, they adding stuff in the middle- what you are not saying. They just adding it, and whatever stuff. -Nepali participant |
| Occasional egregious behavior by interpreters | [The interpreter] just told her, “Don’t talk to them. They just try to make money on you, so you need to talk to them?” That’s what the interpreter told her. Yeah, they made that decision for her. -Karenni Participant |
|  | She told the interpreter to translate for her and the interpreter told her, “Not that. You don’t need to let them know.” So, they probably just blocked her and told her not to tell something. -Karenni Participant |
|  | When he interpreted in-person, he wouldn’t let me ask questions to the doctor. When I asked or tried to ask questions, he became impatient and mean. -Karen Participant |
|  | So they [the family of the patient] want to ask more questions. They want to know, right, and that interpreter tried to stop. “That’s okay.” “Forget about it.” “Don’t ask that so much.” -Karen Participant |
|  | I don’t know, because some of the time he was an interpreter for me two times. And then I don’t like it sometimes. The way that he talks. He’s mean and he doesn’t say the right thing that the doctor says, and then, you know, he doesn’t say the right thing. “Why you guys like that?” “You’re asking too many questions.” “Why are you so naive? Like so dumb.” but he says it in Karen. -Karen Participant |
